# Homecare for sick family members while waiting for medical help during the 2014-2015 Ebola outbreak in Sierra Leone: a mixed methods study

**DOI:** 10.1136/bmjgh-2020-002732

**Published:** 2020-07-21

**Authors:** Kirsten Schmidt-Hellerau, Maike Winters, Padraig Lyons, Bailah Leigh, Mohammad B Jalloh, Paul Sengeh, Alhaji Babah Sawaneh, Zangin Zeebari, Mariano Salazar, Mohamed F Jalloh, Helena Nordenstedt

**Affiliations:** 1 Department of Global Public Health, Karolinska Institute, Stockholm, Sweden; 2 Department of Community Medicine, University of Sierra Leone College of Medicine and Allied Health Sciences, Freetown, Western Area, Sierra Leone; 3 Office of the Chief Executive Officer, FOCUS 1000, Freetown, Sierra Leone; 4 Research and Evaluation, FOCUS 1000, Freetown, Sierra Leone; 5 Jönköping International Business School, Jönköping University, Jonkoping, Sweden; 6 Division of Global Health Protection, Centers for Disease Control and Prevention, Atlanta, Georgia, USA

**Keywords:** viral haemorrhagic fevers, KAP survey, qualitative study, other study design, control strategies

## Abstract

**Introduction:**

Caring for an Ebola patient is a known risk factor for disease transmission. In Sierra Leone during the outbreak in 2014/2015, isolation of patients in specialised facilities was not always immediately available and caring for a relative at home was sometimes the only alternative. This study sought to assess population-level protective caregiving intentions, to understand how families cared for their sick and to explore perceived barriers and facilitators influencing caregiving behaviours.

**Methods:**

Data from a nationwide household survey conducted in December 2014 were used to assess intended protective behaviours if caring for a family member with suspected Ebola. Their association with socio-demographic variables, Ebola-specific knowledge and risk perception was analysed using multilevel logistic regression. To put the results into context, semi-structured interviews with caregivers were conducted in Freetown.

**Results:**

Ebola-specific knowledge was positively associated with the intention to avoid touching a sick person and their bodily fluids (adjusted OR (AOR) 1.29; 95% CI 1.01 to 1.54) and the intention to take multiple protective measures (AOR 1.38; 95% CI 1.16 to 1.63). Compared with residing in the mostly urban Western Area, respondents from the initial epicentre of the outbreak (Eastern Province) had increased odds to avoid touching a sick person or their body fluids (AOR 4.74; 95% CI 2.55 to 8.81) and to take more than one protective measure (AOR 2.94; 95% CI 1.37 to 6.34). However, interviews revealed that caregivers, who were mostly aware of the risk of transmission and general protective measures, felt constrained by different contextual factors. Withholding care was not seen as an option and there was a perceived lack of practical advice.

**Conclusions:**

Ebola outbreak responses need to take the sociocultural reality of caregiving and the availability of resources into account, offering adapted and acceptable practical advice. The necessity to care for a loved one when no alternatives exist should not be underestimated.

**Key questions**

**What is already known?**

- Taking care of someone with Ebola was a major source of infection during the Ebola outbreak in West Africa in 2014/2015.
- Until late in the outbreak, response capacities were insufficient to allow isolation of all patients in specialised facilities. To some degree, information was given to families with a suspected Ebola patient at home on how to protect themselves while waiting for medical help to arrive.
- Homecare interventions have been considered in outbreaks of Ebola and other infectious diseases, including the current COVID-19 pandemic.

**What are the new findings?**

- Ebola-specific knowledge is associated with behavioural intentions to reduce risk of transmission during homecare, and regional differences potentially suggest an association between exposure to the outbreak and protective intentions.
- Reported individual protective caregiving behaviours were constrained by several factors, among them a lack of practical advice matching the caregivers’ reality including the perceived immediate need to care, a lack of resources and social exclusion.
- The impact of an individual’s risk perception of getting Ebola on intended protective caregiving behaviours was limited, which might be explained by the finding that refraining from caregiving was not perceived as an option.

**Key questions**

**What do the new findings imply?**

- Health risk communication needs to take into account that family members do not perceive refraining from caregiving for a sick loved one as an option. Therefore, messages that cannot be aligned with caregiving (for example, to not touch a sick family member) are unlikely to be followed.
- Recommendations need to be adapted to the context to be practical, offering advice that caregivers have the possibility to follow with the resources available to them.

## Introduction

Ebola virus disease (Ebola) is characterised by a high case fatality. During outbreaks, Ebola is transmitted between humans.^1^ The largest epidemic recorded to date occurred in West Africa in 2014/2015. In Sierra Leone, disease transmission occurred in all districts,^2^ over 14 000 cases and almost 4000 deaths were reported.^1^

An important aspect for containing the outbreak was to reduce the number of secondary infections resulting from Ebola patients.^3^ Transmission of Ebola occurs via bodily fluids, and caregiving is a major risk factor for transmission.^1 4^ During the outbreak in West Africa, caring for an Ebola patient was a common way of becoming infected^1 5–7^ and seropositivity prevalence is higher in those with higher exposure to bodily fluids.^8 9^ In Sierra Leone, public campaigns informed about Ebola symptoms, advised to avoid contact with sick people and to call a national toll-free hotline for help.^10^ Suspected Ebola patients were to be transported by ambulance to a specialised facility.^10 11^ However, until late in the outbreak, demand overwhelmed the response infrastructure.^2 10^ Due to these constraints, family members could be left caring for a suspected Ebola patient at home.^2^ Messages addressing these families advised frequent handwashing, separation of the patient from others, avoidance of touching the patient and items in contact with him and assigning one single caregiver.^12–15^ Other reasons for caregiving at home included mistrust of the health system, fear of riding ambulances, misinformation and Ebola-related stigma.^7 16–18^

In previous Ebola outbreaks, there had been limited efforts to support infection prevention and control in households caring for suspected Ebola patients. In 1995 in the Democratic Republic of Congo (DRC), families in remote areas received chlorine, gloves, care-giving advice and follow-up visits.^19^ This approach was operationalised by the WHO in 2003 in the DRC and recommended for situations where no treatment unit is available or when families refuse hospitalisation.^20^ The 2014 WHO recommendations reinforce that this strategy does not offer full protection of transmission, but this is to be preferred rather than to loosing sight of the patient.^20^

In Liberia, a large scale campaign supporting home-care was established during the peak of the 2014 outbreak, while the goal remained to treat every patient in an adequate facility.^21 22^ Considering the various strategies employed in the Ebola outbreak response in West Africa it is not possible to quantify the impact of one specific intervention on the outbreak dynamics.^23^ Nevertheless, several studies from the affected countries suggest that behavioural changes were fundamental for containing the epidemic, community-based interventions had significant effects^21 24 25^ and interrupting transmission within households was complementary to other strategies.^6 26^ Improving safety while caring at home when no specialised facility is available has been seen as one of the major lessons of the outbreak in West Africa.^27^

Health-related behaviours of individuals are influenced by the context. The social-ecological model describes a complex interacting network of factors on a personal, social, community and societal level.^28–30^ A study during the Ebola response in Liberia showed that behaviour change was preceded by changes in beliefs, but constrained by physical, structural, sociocultural and institutional factors.^31^ Understanding of home-based care can aid the development of effective strategies to reduce household transmission. In this study we aimed to obtain a contextual understanding of intended and reported protective measures when caring for suspected Ebola patients at home during the Ebola outbreak in Sierra Leone.

## Methods

A sequential explanatory mixed methods design was used to achieve contextual understanding.^32 33^ First, quantitative population-level survey data collected in Sierra Leone during the Ebola outbreak was analysed to assess protective intentions in the case of caring for a family member falling ill of suspected Ebola and sociodemographic factors associated with such intentions. Second, semi-structured interviews with caregivers of suspected Ebola patients followed to explore how families cared for suspected Ebola patients at home. The interview guide built on the quantitative data by including survey items, aiming to understand the quantitative results more in depth and put them into the context of the caregivers’ reality. The integrated interpretation of the quantitative and qualitative results followed the socio-ecological framework.

### Quantitative phase

#### Design

A cross-sectional Knowledge, Attitudes and Practices (KAP) survey with a total of 3540 respondents was conducted nationwide in Sierra Leone shortly after the peak of the outbreak. At that time, transmission of Ebola was still intense throughout the country except in some southern districts.^34^

#### Sampling

The sampling approach for the KAP survey has been described elsewhere.^35^ In short, multistage cluster sampling was used to randomly select enumeration areas, households and individuals. In addition to each household head, either an adult female (25 years and above) or young person (15 to 24 years) was interviewed.

#### Data collection

Data collection took place in December 2014. The survey was conducted face-to-face in Krio and translated to other local languages by trained data collectors. As part of the questionnaire, intended protective caregiving behaviours were measured by what participants cited unprompted to the question “While waiting for help, how would you care for a family member suspected of having Ebola?”. Recorded were six intended behaviours aimed at reducing Ebola transmission: isolation, using a single caregiver, not touching the sick person, not touching things that the sick person has touched, using protective barriers like gloves and frequent handwashing. Furthermore, the survey collected sociodemographic data, perceived risk of Ebola acquisition within the next 6 months and several items capturing knowledge.

#### Variables

The main outcome was the intention to avoid touching the person or their bodily fluids, as this is the central action that other protective caregiving behaviours are related to. In addition, a score reflecting the number of intended protective caregiving behaviours was created and dichotomised at the mean for analysis (≤1 versus >1).

As independent variables, the sociodemographic factors age, gender and education were assessed. Religion was included as it might affect perception of disease and related behaviours^36^ and because religious leaders were increasingly important stakeholders of health communication during the epidemic.^37^ Further theoretically plausible variables influencing individual health behaviour include knowledge and risk perception.^38^

Eight items were used to measure Ebola-specific knowledge. A composite score was computed and later dichotomised (high vs low) at the mean number of correct answers. A dichotomised variable measuring self-reported perceived risk of getting Ebola was used (no vs any perceived risk) (online supplementary table 1).

10.1136/bmjgh-2020-002732.supp1 Supplementary data

#### Analysis

Multilevel logistic regression analysis was used to account for the hierarchical structure of the data from the individuals clustered in randomised enumeration areas (IBM SPSS Statistics, V.23.0. Armonk, New York: IBM Corp, generalised linear modelling).^39^ For each of the two main outcomes, regression analysis was performed for each independent variable and as a multivariable analysis including all independent variables.

Only complete cases were examined. The number of excluded cases, due to having missing data or lacking inclusion criteria, is negligible (0.5%).

### Qualitative phase

Individual semi-structured interviews were conducted in Freetown in March 2019 with 11 respondents who had been caring for sick relatives at home during the outbreak. Contact with potential participants was facilitated by The Sierra Leone Association of Ebola survivors (SLAES).

Purposive heterogeneous sampling was used to represent a variety of age, education and occupation. Table 1 describes the participants’ characteristics. As per Malterud’s concept of information power,^40^ sample size depends on the amount of information the sample holds regarding the study. Since the study’s aim was relatively narrow and the sample quite specific, 11 participants were deemed sufficient.

**Table 1:** Characteristics of interview participants

Gender, n (%)	
Male	5 (45)
Female	6 (56)
Age years	
Range	25 to 47
Mean (SD)	33.8 (±6.4)
Median	34
Education, n (%) or years	
No formal education	1 (9)
Completed primary schooling*	7 (64)
Completed secondary schooling or above†	3 (27)
Mean years of education	7.9
Occupation, n (%)	
Street vendor	5 (46)
Daily labour	3 (27)
Teacher	1 (9)
Unemployed	2 (18)
Residence, n (%)	
Freetown	9 (82)
Western rural	2 (18)
Religion, n (%)	
Christianity	7 (64)
Islam	4 (36)

*6 years of primary school.

†6 years of secondary school.

The interview guide was constructed based on the behaviours identified in the KAP survey to explore what these behaviours meant to the informants and if and how they were put into practice. It was reviewed for cultural appropriateness and local contextualisation by co-authors who are Sierra Leonean and have experience conducting qualitative assessments in Sierra Leone (MFJ and PS) and by senior members of the SLAES. Data were collected through face-to-face interviews at the head office of the SLAES. All participants preferred being interviewed in Krio. The interviews were conducted by a local researcher (ABS) with experience in qualitative data collection. The length of the interviews was about half an hour. All audio-recordings were transcribed by the interviewer, complete and verbatim directly from Krio into English.

Analysis was conducted using the web-based programme Dedoose for managing qualitative data. Thematic analysis, as described by Braun and Clarke,^41^ was used to analyse the data. Familiarisation with the data included reading the transcripts several times and discussing them with the interviewer. As little previous knowledge exists on the topic, an inductive approach to coding was chosen. The codes of the initial four interviews were in the end re-reviewed for consistency. Overarching themes were developed by analysing the codes and comparing back to the data. They were further refined by evaluating them vertically within each interview and horizontally across the data set. This interpretive process was tracked by memo writing.^42 43^

### Patient and public involvement

Patients or public were not involved in the design and conduct of this study.

The committee (Sierra Leone Ethics and Scientific Review Committee) considers survey participants 15 years or older capable to consent. Written or thumb-printed informed consent was obtained from each participant.

## Results

In total, 3600 potential participants were approached, of which 98% (3540) agreed to participate in the survey. Their sociodemographic characteristics are listed in table 2.

**Table 2:** Sociodemographic characteristics of the KAP survey participants*

	Northern province	Eastern province	Southern province	Western area	Total
	N (%)	N (%)	N (%)	N (%)	N (%)
Age					
15–20	277 (22)	227 (25)	152 (27)	168 (21)	824 (23)
21–35	386 (31)	303 (33)	172 (31)	284 (35)	1145 (33)
36–49	275 (22)	206 (22)	120 (22)	199 (25)	800 (23)
50 or older	298 (24)	182 (20)	113 (20)	157 (19)	750 (21)
Gender					
Male	665 (54)	472 (51)	261 (47)	399 (49)	1797 (51)
Female	571 (46)	446 (49)	296 (53)	409 (51)	1722 (49)
Education					
No formal education	545 (44)	343 (37)	149 (27)	151 (19)	1188 (34)
Some primary/primary	238 (19)	234 (26)	98 (18)	97 (12)	667 (19)
Secondary or above	453 (37)	341 (37)	310 (56)	560 (69)	1664 (47)
Religion					
Islam	1054 (85)	535 (58)	323 (58)	412 (51)	2324 (66)
Christianity	182 (15)	383 (42)	234 (42)	396 (49)	1195 (34)
Total	1236 (35)	918 (26)	557 (16)	808 (23)	3519 (100)

*The total sample was 3540 but we restricted to only show respondents included in the complete case analysis. Respondents with one or more missing values (n=21) have been excluded.

KAP, Knowledge, Attitudes and Practices.

### Sociodemographic factors, Ebola-specific knowledge, risk perception and intended protective caregiving behaviour

**Figure 1:**
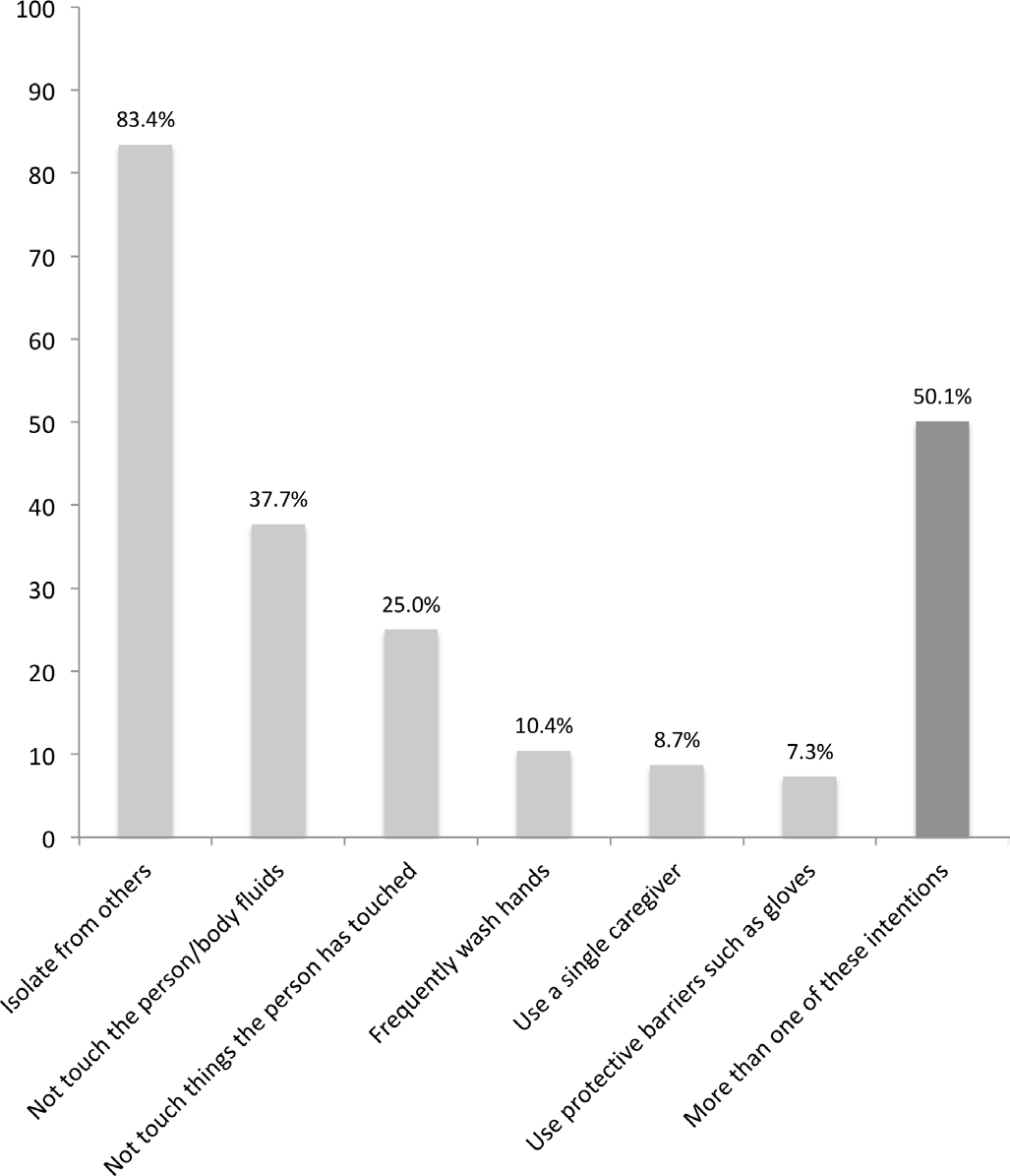
Percentage of survey participants reporting various protective caregiving behaviours they intend to use to stay safe while caring for suspected Ebola patients.

Almost half of the respondents (43%) perceived themselves as being at risk of getting Ebola in the next 6 months. Two-thirds (67%) were classified as having higher Ebola-specific knowledge (online supplementary table 2). Of the six intended caregiving behaviours registered by the survey, the mean number stated was 1.7, half the participants (50%) mentioned at least two (data not shown). Figure 1 shows the crude percentage of participants stating each intended protective caregiving behaviour.

The province of residence was the only demographic variable associated with protective intentions to avoid touching a sick person and their bodily fluids (Reference: Western Area. Northern Province: adjusted OR (AOR) 2.06, 95% CI 1.14 to 3.61; Eastern Province: AOR 4.74, 95% CI 2.55 to 8.81). Higher Ebola-specific knowledge was positively associated with increased odds of intending to avoid touching a sick person or their bodily fluids (AOR 1.29, 95% CI 1.01 to 1.54). Perceiving oneself at risk was significantly negatively associated with this outcome (AOR 0.79, 95% CI 0.67 to 0.93). Two of these factors were also positively associated with the second outcome, stating more than one intended protective caregiving behaviour: province of residence (AOR 2.94; 95% CI 1.37 to 6.34) and higher Ebola-specific knowledge (AOR 1.38; 95% CI 1.16 to 1.63) (table 3). For both outcomes, univariable results were very similar to adjusted results.

**Table 3:** Associations between sociodemographic factors, knowledge and risk perception and intended protective caregiving behaviour

	Expressing the intention to not touch the sick person or their bodily fluids			Expressing the intention to take more than one preventive behaviour		
	N (%)	Adjusted OR* (95% CI)	P value	N (%)	Adjusted OR* (95% CI)	P value
Age						
15–20	324 (39.3)	1.0 (Reference)		427 (51.8)	1.0 (Reference)	
21–35	430 (37.6)	0.93 (0.75 to 1.15)	0.481	579 (50.6)	1.01 (0.81 to 1.25)	0.952
36–49	285 (35.6)	0.83 (0.65 to 1.04)	0.110	400 (50.0)	0.97 (0.77 to 1.23)	0.807
50+	289 (38.5)	0.95 (0.74 to 1.21)	0.656	357 (47.6)	0.92 (0.71 to 1.18)	0.492
Gender						
Male	667 (37.1)	1.0 (Reference)		890 (49.5)	1.0 (Reference)	
Female	661 (38.4)	1.03 (0.88 to 1.22)	0.691	873 (50.7)	1.05 (0.89 to 1.24)	0.539
Education						
No formal	468 (39.4)	1.0 (Reference)		567 (47.7)	1.0 (Reference)	
Some primary/primary	275 (41.2)	1.01 (0.80 to 1.28)	0.917	352 (52.8)	1.08 (0.85 to 1.37)	0.519
Secondary or above	585 (35.2)	0.88 (0.72 to 1.07)	0.205	844 (50.7)	1.18 (0.96 to 1.45)	0.113
Religion						
Islam	889 (38.3)	1.0 (Reference)		1168 (50.3)	1.0 (Reference)	
Christianity	439 (36.7)	1.05 (0.87 to 1.26)	0.619	595 (33.7)	1.15 (0.96 to 1.39)	0.082
Region						
Western area	199 (24.6)	1.0 (Reference)		341 (42.2)	1.0 (Reference)	
Northern province	444 (35.9)	2.06 (1.14 to 3.61)	0.017	609 (49.3)	1.46 (0.70 to 3.01)	0.311
Eastern province	495 (53.9)	4.74 (2.55 to 8.81)	0.000	580 (63.2)	2.94 (1.37 to 6.34)	0.006
Southern province	190 (34.1)	1.80 (0.90 to 3.71)	0.099	233 (41.8)	0.92 (0.39 to 2.16)	0.832
Ebola-specific knowledge						
Lower	358 (31.5)	1.0 (Reference)		481 (42.3)	1.0 (Reference)	
Higher	970 (40.7)	1.29 (1.01 to 1.54)	0.004	1282 (53.8)	1.38 (1.16 to 1.63)	0.000
Risk perception						
No	793 (39.6)	1.0 (Reference)		1004 (50.2)	1.0 (Reference)	
At least some	535 (35.2)	0.79 (0.67 to 0.93)	0.006	759 (50.0)	1.15 (0.97 to 1.37)	0.100

*Adjusted for age, gender, education, religion, region, Ebola-specific knowledge and risk perception.

### Reported caregiving behaviour and caregivers’ perceptions

The qualitative findings allowed to expand understanding of giving care to a suspected Ebola patient at home. The first of four themes refers to the immediate need to care for a loved one who is suffering. Participants often reported ambivalence to the perceived helpfulness of the health system and the Ebola response, which constitutes the second theme. The last two themes draw a wider circle around the individual, which is constrained by unpreparedness and a lack of resources and affected by social disruption and exclusion. Table 4 shows themes and subthemes.

**Table 4:** Themes and subthemes of the qualitative analysis

Theme	Subthemes
The immediate need to care	Caring is not optional
	Helping the sick person
	Knowing the risk and taking precautions
Ambivalent perception of the health system and its capacities	Medical help as a solution
	Health system/Ebola response do not provide a solution
	Fearing separation
Unpreparedness and lack of resources	Did not take it for Ebola initially
	Receiving information
	Financial constraints and lack of resources
Social disruption and exclusion	Negative impact on family structures
	Fear leads to exclusion
	Fear and preventive measures during the outbreak impact society

#### The immediate need to care

All participants related to the self-evidence of giving care to a family member in urgent need. For them, when a loved one fell ill, giving care was not something one decided to do or not to do, but rather an immediate and obvious necessity that was not questioned.

‘They said we should wash our hands, we should not touch a dead body, we should not touch a sick person. […] But when my children have gotten sick, nobody did come and take them. […] I made up my mind not to leave my children. […] I did not refuse to touch my daughter. […] Because she was my blood I can’t leave her suffering.’ (Participant 04)

Caregivers tried to help as well as they could, even though all of them were aware of the transmission risk involved in caregiving and worried about their own and others’ safety. Many talked about taking this risk deliberately.

‘I said that if it caused me to die for the sake of my aunt, I will do so. […] She is my aunt and I loved her, she is the only hope I have.’ (Participant 11)

All participants reported some way they had tried to reduce the risk of transmission, usually not by omitting measures of caregiving, but rather by trying to protect themselves while giving care. All respondents had heard and accepted at least some recommendations that were given to prevent Ebola transmission, but they did not feel able to follow the advice, or even consciously decided against adhering to the advice. They felt that especially the recommendation to not touch the patient could not be integrated into the reality of needing to provide care.

‘They were saying you should not touch, but since she was my wife and she gave birth for me, so it is a culture for me. […] I loved her so much. […] So when that happened to her I decided to turn a deaf ear. I did not even care about what the government was saying, because it is life.’ (Participant 08)

The recommendation to keep away from a patient was generally well known and most participants mentioned ways in which they had tried to obtain more distance. For instance, several caregivers tried to establish some physical separation within the same room by not sharing the same bed. Several respondents voiced specific concerns regarding children, trying to keep them away from the sick person. Regarding handwashing, participants reported a wide array of behaviours ranging from regularly using chlorine to not washing hands at all. Only few participants mentioned having used gloves or plastic bags for their hands for protection, for example, when washing soiled clothes. Almost all participants mentioned strategies to deal with objects that were soiled or in contact with the sick person. Washing clothes and keeping items separated were commonly cited, as was burning items, including mattresses.

#### Ambivalent perception of the health system and its capacities

Medical care was widely seen as potentially beneficial for the patient by the respondents, but at the same time all were critical towards how much help there is to be expected from the health system and sceptical about quality of care.

‘At that time, when going to the hospital, to see a nurse is a problem. The nurse will be afraid of you, and you will also be afraid of the nurse, everyone was afraid of each other.’ (Participant 05)

Many recalled waiting for the medical help they had called for, but it arrived too late, or never. For many, professional medical care did not seem to be an option available to them.

‘I should have taken her to the hospital on that day. But at that time the hospitals were not functioning.’ (Participant 01)

#### Unpreparedness and lack of resources

The main health messages promoted by the outbreak response were generally well known. Many caregivers explained how they were able to recognise the disease as Ebola. Almost all participants recounted several recommended measures of protecting themselves, commonly including not touching sick persons. Nevertheless, all caregivers felt unprepared and not sufficiently informed about what they could do in their specific situation. Some recommendations were perceived as not applicable and insecurity remained around how to practically follow the advice.

‘I was trying to help as a mother, but I did not have someone that can help or advise, so what comes to my mind was what I was doing.’ (Participant 04)

In addition, all interviewees reported some form of financial constraints. Underlying poverty meant a lack of living space, relying on water taps outside the residence and few monetary reserves to compensate for loss of income and cover of additional expenses.

‘When my daughter got sick all my business collapsed. All my money went to the hospital.’ (Participant 04)

#### Social disruption and exclusion

Most respondents experienced several cases of Ebola within the family. Disease and death often led to changed roles and responsibilities within families. In two cases, tensions within the family were hinted at. One was related to a sick husband disagreeing with his wife trying to keep the children away from him for their safety, and one to the socially assigned role of being a caregiver.

‘She was a sister of my husband, I didn’t want to destroy my marital home, my mind was striking that it might be Ebola.’ (Participant 01)

One of the most dominant topics was social exclusion. All respondents mentioned several ways in which they were affected. Not only neighbours and friends, but also family members distanced themselves, the caregiver felt alone and without support.

‘Everybody you did not expect to was turning against you. People would not walk close to you, they would not visit you. […]People that we trusted turned against us.’ (Participant 10)

Commonly, people in the caregiver’s surroundings were perceived as talking negatively about or to them. Several mentioned movement restrictions imposed on the caregiver by neighbours, many including reduced access to water. Some participants also reported that even though they had money, they could not access food or other items, because nobody would want to come near them or take their money.

‘During that time my neighbours, they started pointing their fingers at us, they said I am an Ebola family. If we went to buy anything, they wouldn’t hold the money. […] The neighbours segregated themselves from us, even the well where we used to fetch water, they stopped coming there because we were there.’ (Participant 06)

## Discussion

Apart from describing intended and reported protective caregiving behaviours when caring for a suspected Ebola patient at home, several factors influencing these behaviours could be identified on all levels of the socio-ecological model. Province of residence and Ebola-specific knowledge were shown to be associated with intended protective behaviour. Among the most important perceived barriers to putting protective intentions into practice was the unquestioned and immediate necessity to care for a sick loved one, that went beyond rationalising the risk of Ebola acquisition. Furthermore, possibilities were limited by the physical environment and a lack of resources. Therefore generalised messages were not found to be practical to adhere to.

### Protective caregiving behaviours

All intended protective caregiving behaviours captured by the KAP survey were communicated to the public at some point during the outbreak.^14 44 45^ However, comprehensive data specifying which exact messages were spread when, where and how, are lacking. Some more specific advice for households was mentioned in the interim recommendations of the Sierra Leone Emergency Operations Committee in October 2014, and a detailed concept for caring at home can be found in a WHO guide for outbreaks of Ebola and Marburg virus from August 2014, but it is unclear to what extent these messages were easily available to the public.^20 45^

Survey participants most commonly stated one to three intended measures to protect themselves in the case of a family member falling ill with suspected Ebola. Most commonly mentioned were isolation, not touching the person or their bodily fluids and not touching the things the person has touched. KAP survey results from Liberia later in the outbreak showed similar results, except for a higher proportion intending to wash hands (16% compared with 10%) and to use gloves or plastic bags as protective equipment(37% compared with 7%).^46^ This difference could potentially be explained by the strategy implemented in Liberia to improve homecare.

Qualitative interviews confirmed that in practice, all caregivers have striven to take at least some measures to protect themselves, which were mostly aligned with public recommendations. In accordance with the KAP survey results, especially the recommendation not to touch a sick person was reported to be well known. However, none of the interviewees recounted strictly following this recommendation. Similarly, while many tried to maintain physical distance, none of the behaviours described would equal what would be considered isolation from a biomedical point of view. It also became clear that the concept of isolation, which was stated as an intention by the majority in the survey, was understood in different ways and that circumstances inhibited practical implementation. Handwashing was neither a common intention nor a commonly reported behaviour. This was partly due to the limited accessibility of water, soap and chlorine, but it also did not seem to be a behaviour prioritised by the participants.

### Factors influencing caregiving behaviour on an individual level

When controlled for Ebola-specific knowledge, regional differences persisted: Residing in the Eastern province was significantly associated with both protective outcomes. One difference between the regions at the time of quantitative data collection was their different level of the outbreak. The epidemic started to gain a foothold in the Eastern Province in May 2014,^2^ and by the time the situation there began to stabilise in September 2014,^2^ cases had started to build up in the Northern Province and Western Area at the time of the KAP survey.^47^ Therefore, the regional differences suggest that the duration and intensity of being exposed to the outbreak possibly correlate with intended protective caregiving behaviours in a way independent of the amount of information received. It has been previously suggested that experiencing Ebola cases in a community leads to increased acceptance of Ebola, behaviour change and rapid social learning.^31 48^

Ebola-specific knowledge was significantly associated with intended protective caregiving behaviour in quantitative analysis. Accordingly, in the qualitative phase, interviewees reported hearing Ebola messages and described how they, to some extent, affected their intentions and behaviours. However, there was a strong feeling of lacking appropriate advice for the specific situations they found themselves in. For example, what can be done when the call for medical help is not answered and a loved one urgently needs care or transport that cannot be given without touch? A qualitative study conducted in Liberia with community leaders in September 2014 came to similar conclusions: basic messages were understood and accepted, but the question of how to put them into practice in the given context remained, and messages did not sufficiently address the need for physical contact in certain situations.^49^

Crucial for understanding caregiving behaviours is that caring for a loved one was not perceived as optional and the risk involved was taken consciously. This has been described previously in a qualitative study regarding caregivers’ situations during the 2000/2001 Ebola outbreak in Gulu, Uganda.^50^ Similarly, members of the Ebola Response Anthropology Platform commented that knowledge of the biomedical risk might be of limited importance in caregiving situations.^51^ It is one of many potential factors explaining the inconsistent survey findings regarding risk perception.^52^

In line with our results, a study evaluating Ebola prevention promotion in the Gambia showed an association between Ebola-specific knowledge and the intention to avoid touching a sick person, and reasons respondents gave as to why they would touch a suspected patient included need, willingness, duty and a family member becoming sick.^53^

Another barrier for protective caregiving behaviour was a lack of resources, further exacerbated by a negative impact on income due to one household member being sick and caregiving by another household member. Poverty affected living conditions, access to water and affordability of items like gloves and chlorine, and disposal of soiled items requires their replacement.

### Factors influencing caregiving behaviour on a community level

On a community level, the most important factor influencing caregiving behaviour was social exclusion. Caregiver and patient were likely to remain isolated and with little support. Negative reactions of others also led to limited access to essential resources like food and water. Discrimination within their communities, up to being rejected by their community, is a problem recurrently reported for healthcare workers in Sierra Leone, but also in other Ebola outbreaks.^7 17 19 54^

### Factors influencing caregiving behaviour on a societal level

During the qualitative interviews, access to water was a recurring topic, mostly because of restricted access to it due to fears of the neighbours. But it needs to be considered that there is an underlying insufficient access to water affecting most Sierra Leoneans. In Western Area Urban, for example, where interviewees were recruited, less than 4% of the population have piped water at home and an additional 17% have access to piped water in their compound, while all others depend on sources like public taps and wells.^55^

Census data from 2015 show that about one-third of the households occupied only one room, in urban areas this proportion increased to almost 40%.^55^ Some participants referred to crowded living conditions and attempts to achieve physical separation from the sick person often took place within the same room. In light of this, recommendations and intentions to isolate might be understood in very different ways and not be seen as, or not physically be, feasible.

### Strengths and limitations

A major strength of this study is the combined methodology, using results from both a nationwide survey and from interviews with those personally affected. It gives insights into a topic that has not yet been previously studied, including how knowledge and intentions translate into specific situations. Nevertheless, there are limitations that need to be considered.

While KAP surveys are widely used and have many advantages, they also have limitations. Among them is the risk of questions being understood differently by different people, due to underlying linguistic and cultural issues.^56 57^ To mitigate this limitation, KAP questions were developed by an experienced team of Sierra Leoneans and pilot-tested.

Measuring knowledge by of a score of eight chosen questions and dichotomising at the mean is arbitrary, but considering that there is no validated score, this definition follows a previous publication of this KAP data.^58^ Also, it is unclear to what extent the stated behavioural intentions were related to actual caregiving behaviour. Especially when evaluating practices, there is a risk of measuring the behavioural norm and/or which information reached participants rather than practices.^59^ Using a mixed methods approach addressed some of these limitations by using qualitative data to understand quantitative results more in depth and to add contextual understanding.

Qualitative interviews were conducted more than 4 years after the outbreak, which might have led to omission of details. However, this should be mitigated by the fact that the experience of caring for a loved one during an outbreak is likely to be something that has a high chance of being remembered. Participants were all interviewed in Freetown. Given that socio-cultural factors and physical living environment might be quite different in rural areas and differ between regions, findings are limited to the capital.

## Conclusion

Our results underline the importance of carefully adapting health communication and interventions to sociocultural and material realities. Knowledge is an important factor facilitating protective behaviour, but there was a perceived lack of acceptable practical advice suitable for the specific situation the caregivers found themselves in. Messages as broad and as impactful to caregiving as not to touch a sick person were of questionable use, as the need to care for a loved one cannot be dismissed. Because caregiving is not optional but an unquestioned necessity, it is not subject to rational risk assessment, and messages targeting risk awareness without acknowledging this might have limited effects. Furthermore, actions were limited by the circumstances: Where several people live in a single room, separating a sick person from others may not be easily feasible, and access to water and other resources may be limited. Recommendations and interventions need to take the reality of caring and availability of resources into account to be accepted and put into practice. Adapting health risk communication to local circumstances seems as important for other infectious disease outbreaks as it is for Ebola. Social distancing as part of the response to coronavirus disease (COVID-19) faces similar challenges regarding lack of physical living space and material resources in many settings.

An important area for future research is the implementation and evaluation of possible approaches supporting homecare situations in Ebola outbreaks when appropriate isolation facilities cannot be accessed or are not easily accepted.
