# Supplementary material for: Homecare for sick family members while waiting for medical help during the 2014-2015 Ebola outbreak in Sierra Leone: a mixed methods study
Source: BMJ Glob Health. 2020 Jul 21;5(7):e002732. doi: 10.1136/bmjgh-2020-002732 (PMC7375393; doi:10.1136/bmjgh-2020-002732)
Supplement: Supplementary data [file bmjgh-2020-002732supp001.pdf]

Supplementary Table 1: Independent and dependent variables and their classifications

Domain	Variables	Value
**Independent Variables**		
Socio-demographic	Age	15-20<br>21-35<br>36-49<br>50+
	Gender	Male<br>Female
	Educational Level	No education<br>Some primary/primary<br>Secondary and above
	Religion	Islam<br>Christianity
	Region	Northern province<br>Eastern province<br>Southern province<br>Western area
Ebola specific Knowledge	Viral cause of Ebola: Yes	Below the mean: 0-6 correct<br>Above the mean: 7-8 correct
	Bats and monkeys as cause of Ebola: Yes	
	Avoiding burial rituals is protective: Yes	
	Treatment in facility increases survival: Yes	
	Treatment in facility reduces spread: Yes	
	Traditional healers can cure Ebola: No	
	Spiritual healers can cure Ebola: No	
	Patient is cared for at the hospital: Yes	
Risk perception	No risk<br>Small risk<br>Moderate risk<br>Great risk<br>I don't know/not sure	No: No risk perception, don't know/not sure<br>Yes: Any risk perception
**Outcome Variables**		
Number of intended preventive behaviours	Intention to isolate	Below the mean: 0-1<br>Above the mean: 2-6
	Intention to use single caregiver	
	Intention to not touch the person or their bodily fluids	
	Intention not to touch things the person has touched	
	Intention to use protective barriers	
	Intention to frequently wash hands	
Not touching	Intention to not touch the person or their bodily fluids	No<br>Yes

Supplementary table 2: Frequencies of original and composite independent variables

	n	%
Virus causes Ebola	2156	61.3
Bats/monkeys/other wild animals cause Ebola	2803	79.7
Preventable by avoiding burial rituals involving touching the body of someone who died of Ebola	3393	96.4
Immediate treatment in health facility increases chances of survival	3353	95.3
Immediate treatment in health facility reduces chance of spreading	3239	92.0
Refuting Can be successfully treated by spiritual healers	3080	87.5
Refuting Can be successfully treated by traditional healers	3293	93.6
Somebody going to the health facility with Ebola will receive care	2590	73.4
Higher information	2382	67.7
No risk	1922	54.6
Small risk	793	22.5
Moderate risk	289	8.2
Great risk	436	12.4
I don’t know/not sure	79	2.2
At least small risk perception	1518	43.1
